# Pioglitazone Reduces Dementia Risk in Patients with Type 2 Diabetes Mellitus: A Retrospective Cohort Analysis

**DOI:** 10.3390/jcm7100306

**Published:** 2018-09-27

**Authors:** Chin-Hsiao Tseng

**Affiliations:** 1 Department of Internal Medicine, National Taiwan University College of Medicine, Taipei 10051, Taiwan; ccktsh@ms6.hinet.net; 2 Division of Environmental Health and Occupational Medicine, National Health Research Institutes, Zhunan 350, Taiwan

**Keywords:** dementia, diabetes mellitus, pioglitazone, Taiwan

## Abstract

Background: The beneficial effect of pioglitazone on dementia requires confirmation. Methods: The database of Taiwan’s National Health Insurance was used to enroll a propensity score-matched-pair cohort of patients who had ever used pioglitazone and patients who had never used pioglitazone from Taiwanese patients with newly diagnosed diabetes mellitus during 1999–2008. The patients were to be alive on 1 January 2009 and were followed up for dementia until 31 December 2011. Hazard ratios were estimated using the Cox proportional hazards model. Results: There were 11,011 never users and 11,011 ever users of pioglitazone, with respective numbers of incident dementia of 123 and 91. The overall hazard ratio was 0.716 (95% confidence interval: 0.545–0.940) for ever users versus never users. The hazard ratios for the first (<11.0 months), second (11.0–19.6 months) and third (>19.6 months) tertiles of cumulative duration were 0.806 (0.544–1.193), 0.654 (0.430–0.994) and 0.694 (0.469–1.026), respectively. When cumulative duration was treated as a continuous variable, the hazard ratio was 0.987 (0.976–0.998). In subgroup analyses, the beneficial effect was mainly observed in patients who had not been treated with metformin. Among metformin ever users, the hazard ratio for dementia for pioglitazone ever users versus never users was 0.802 (0.580–1.109); and was 0.494 (0.284–0.857) among never users of metformin. No interaction between pioglitazone and major risk factors of dementia (i.e., stroke, hypoglycemia, head injury and Parkinson’s disease) was observed. Conclusions: Pioglitazone use is associated with a lower risk of dementia, especially when it is used in never users of metformin and has been used for more than 20 months.

## 1. Introduction

Dementia is characterized by progressive deterioration of memory and can result from either a vascular etiology or a neurodegenerative disease known as Alzheimer’s disease. Alzheimer’s disease has been called “type 3 diabetes” because of the close link between diabetes mellitus and the potential pathophysiological mechanism of brain insulin resistance leading to its development [1]. Patients with diabetes may also have an increased risk of dementia resulting from the increased deposition of advanced glycation end-products, dysregulation of lipid metabolism, atherosclerosis and augmented inflammation, and oxidative stress [2]. Amyloid beta (Aβ) is formed by cleaving the amyloid precursor protein (APP) by secretases [3] and important pathological changes in the brain of patients with Alzheimer’s disease include increased deposition of Aβ and hyperphosphorylation of Tau protein [1].

Insulin sensitizers targeting insulin resistance in the brain can therefore be potentially beneficial in the prevention of Alzheimer’s disease or dementia [1]. An early study using transgenic mice suggested that an acute 7-day oral treatment with pioglitazone or ibuprofen significantly reduced glial inflammation and Aβ levels together with a decreased expression of β-secretase-1 (or β-site APP-cleaving enzyme 1 (BACE1)) [4]. Another recent animal study showed that anti-inflammatory treatment with pioglitazone or interleukin-1 receptor antagonist rescued neuroinflammation in preclinical stages of Alzheimer’s disease [5]. A cellular study confirmed that pioglitazone, by reducing the expression of BACE1, reduced Aβ levels [6]. Additionally, an active component of ginseng, ginsenoside Rg1, may translocate peroxisome proliferator-activator receptor gamma (PPARγ) from cytoplasm to nucleus, and suppress BACE1 activity like pioglitazone (a PPARγ agonist). Such activity was attenuated by the treatment of a PPARγ antagonist [6]. On the other hand, a more recent cellular study showed that MH84 (a novel class of γ-secretase modulator with a function of PPARγ activation), but not pioglitazone, decreased Aβ levels and improved mitochondrial function [7]. Therefore, whether pioglitazone may reduce the risk of Alzheimer’s disease or dementia requires further investigation.

Three preliminary clinical trials suggested contradictory outcomes [8], and a global phase III trial (TOMMORROW) evaluating whether pioglitazone 0.8 mg SR could delay the onset of mild cognitive impairment in high risk individuals has recently been terminated because of the disappointing interim futility analyses [9].

Observational studies evaluating the effect of pioglitazone on dementia risk are also sparse and require confirmation and clarification. Lu et al. compared the risk of dementia in diabetes patients who used pioglitazone as a second-line therapy after metformin with patients treated with other second-line antidiabetic drugs after metformin [10]. They found that patients with dual therapy of pioglitazone and metformin might have a lower risk compared to patients treated with dual therapy of sulfonylureas and metformin. As metformin use is associated with a lower risk of dementia [11], and sulfonylureas [12] and hypoglycemia [13] may increase the risk, several issues related to the design of this recent observational study require additional clarification before pioglitazone can be claimed to exert a beneficial effect on dementia. First, there could be a significantly lower risk of hypoglycemia associated with the combination of pioglitazone and metformin than with the combination of a sulfonylurea and metformin. Therefore, the higher risk of dementia associated with the combination of a sulfonylurea and metformin in the reference group (either because of the higher risk of dementia associated with the sulfonylurea used or because of a secondary effect resulting from a high incidence of hypoglycemia associated with the sulfonylurea used) might have explained the “beneficial” effect observed in the group who used the combination of pioglitazone and metformin (even though pioglitazone might have had a neutral effect only). Second, the case numbers of dementia in the study were too small to allow subgroup analyses or to evaluate a dose-response relationship by treating pioglitazone exposure as a continuous variable. Third, this study did not consider the potential confounding from some important risk factors of dementia such as hypoglycemia, head injury and Parkinson’s disease, even though the effect of stroke had been considered.

The purposes of the present study were to clarify whether pioglitazone could be preventive for dementia in a dose-response pattern and to evaluate whether such an effect, if present, could be independent of metformin and some common risk factors of dementia including stroke, hypoglycemia, head injury and Parkinson’s disease.

## 2. Materials and Methods

The National Health Insurance (NHI), a unique healthcare system covering >99.6% of Taiwan’s population and having contracts with all in-hospitals and 93% of all medical settings, has been implemented in Taiwan since March 1995. The reimbursement records including disease diagnoses, medication prescriptions and performed procedures can be used for academic research. The present study retrospectively analyzed a 1:1 propensity score (PS)-matched cohort derived from the NHI database after ethics approval from the National Health Research Institutes (number 99274).

The database was described in more detail in previously published papers [14,15]. According to the International Classification of Diseases, Ninth Revision, Clinical Modification (ICD-9-CM), diabetes was coded 250.XX and dementia was coded as abridged codes of A210 or A222, or as ICD-9-CM codes of 290.0, 290.1, 290.2, 290.4, 294.1, 331.0–331.2, or 331.7–331.9.

**Figure 1:**
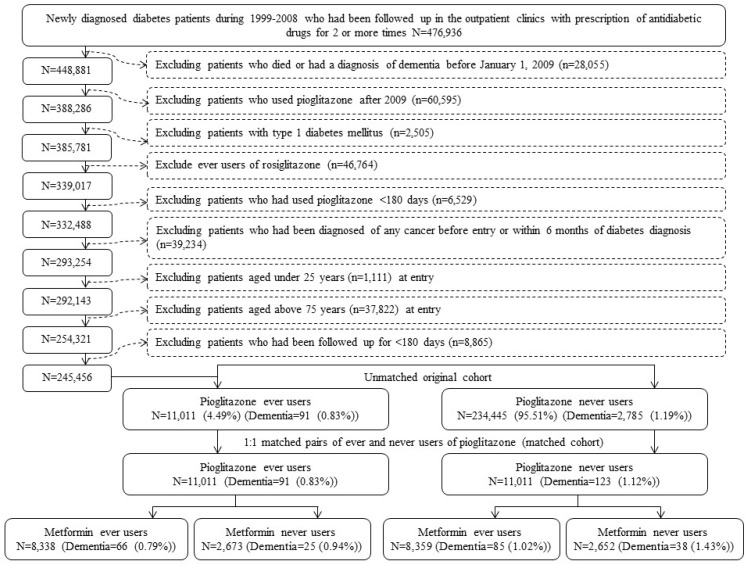
Flowchart for the procedures in selecting a propensity score matched cohort of pioglitazone ever users and never users.

The procedures used to create a matched cohort are shown in Figure 1. First, 476,936 patients were identified from the outpatient clinics with newly diagnosed diabetes during the years 1999–2008. The patients should have been prescribed antidiabetic drugs at least twice in the outpatient clinics. Patients with a diagnosis of diabetes between 1996 and 1998 were not included to ensure a new diagnosis after 1999. The following steps were applied to exclude ineligible patients: (1) patients who died or had a diagnosis of dementia before 1 January 2009 (*n* = 28,055); (2) patients who were initiated with pioglitazone use after 2009 (*n* = 60,595); (3) type 1 diabetes mellitus (*n* = 2505); (4) ever users of rosiglitazone (*n* = 46,764); (5) pioglitazone use for < 180 days (*n* = 6529); (6) diagnosis of any cancer before entry or within 6 months of diabetes diagnosis (*n* = 39,234, cancer patients might have a shortened lifespan and were excluded because they might have distorted follow-up time and dementia could be misdiagnosed from the clinical presentations of malignancy); (7) age < 25 years (*n* = 1111); (8) age > 75 years (*n* = 37,822) and (9) follow-up duration < 180 days (*n* = 8865). As a result, 11,011 ever users and 234,445 never users of pioglitazone were identified (the unmatched original cohort). A matched-pair cohort of 11,011 ever users and 11,011 never users (the matched cohort) was then created by matching on PS based on the Greedy 8 → 1 digit match algorithm [16]. PS was created by logistic regression with all characteristics listed in Table 1 being treated as independent variables. This matching method has been described in more detail in previous studies [14,15]. Among the matched cohort, 16,697 (8359 never users and 8338 ever users of pioglitazone) were ever users of metformin and 5325 (2652 never users and 2673 ever users of pioglitazone) had never been treated with metformin.

The cumulative duration of pioglitazone therapy was calculated in months. Potential confounders included the following categories: demographic data, major comorbidities associated with diabetes mellitus, diabetes-related complications, other major risk factors of dementia, potential risk factors of cancer, antidiabetic drugs, and medications commonly used in diabetes patients. The category of demographic data included age, sex, diabetes duration, occupation and living region (classified as Taipei, Northern, Central, Southern, and Kao-Ping/Eastern). Occupation was classified as class I (civil servants, teachers, employees of governmental or private businesses, professionals and technicians), class II (people without a specific employer, self-employed people or seamen), class III (farmers or fishermen) and class IV (low-income families supported by social welfare, or veterans). The ICD-9-CM codes for hypoglycemia included 251.0, 251.1 and 251.2; and the codes for other potential confounders including major comorbidities associated with diabetes mellitus (i.e., hypertension, dyslipidemia and obesity), diabetes-related complications (i.e., nephropathy, eye disease, stroke, ischemic heart disease and peripheral arterial disease), other major risk factors of dementia (i.e., head injury and Parkinson’s disease) and potential risk factors of cancer (chronic obstructive pulmonary disease, tobacco abuse and alcohol-related diagnoses) can be found in a previously published paper [11]. Antidiabetic drugs included insulin, sulfonylureas, metformin, meglitinide and acarbose; and commonly used medications in diabetes patients included angiotensin converting enzyme inhibitors/angiotensin receptor blockers, calcium channel blockers, statins, fibrates and aspirin.

The standardized difference was calculated for each covariate and a value >10% was used to indicate potential confounding from the variable as proposed by Austin and Stuart [17].

The incidence density of dementia was calculated with regard to the use of pioglitazone in the following subgroups: Never users, ever users and the first (<11.0 months), second (11.0–19.6 months) and third (>19.6 months) tertiles of cumulative duration. The case number of newly diagnosed dementia identified during follow-up was the numerator. The denominator was the follow-up duration in person-years, which started on 1 January 2009 and ended on 31 December 2011, at the time of a new diagnosis of dementia, or on the date of death or the last reimbursement record.

The hazard ratios and their 95% confidence intervals for ever users of pioglitazone and for each tertile of cumulative duration in reference to never users were estimated using the Cox proportional hazards regression model. Additionally, hazard ratios were also estimated for the cumulative duration of pioglitazone therapy being treated as a continuous variable. To further examine whether the effect of pioglitazone could be independent of metformin use, the above analyses were also performed in subgroups of patients of ever and never users of metformin.

To evaluate the joint effects of pioglitazone and major risk factors of dementia (i.e., stroke, hypoglycemia, head injury and Parkinson’s disease), hazard ratios were also estimated in the following four subgroups with regard to the presence and absence of risk factors and pioglitazone use, i.e., (1) risk factor (+)/pioglitazone (−) as the reference group; (2) risk factor (+)/pioglitazone (+); (3) risk factor (−)/pioglitazone (−); and (4) risk factor (−)/pioglitazone (+). The values of *P*-trend and *P*-interaction were also estimated in each model.

The analyses were conducted using SAS statistical software, version 9.3 (SAS Institute, Cary, NC, USA). *p* < 0.05 was considered statistically significant.

## 3. Results

The characteristics of never and ever users of pioglitazone are shown in Table 1. None of the variables had a standardized difference >10%, suggesting that the two groups were well matched in covariates.

Table 2 shows the incidence rates and hazard ratios of dementia by pioglitazone exposure. The overall hazard ratio (0.716, 95% confidence interval: 0.545–0.940) suggested a significantly lower risk of dementia associated with pioglitazone use. In the tertile analyses, a significant *p*-value was observed in the second tertile, while the *p*-value for the third tertile was borderline significant and that for the first tertile was not significant. When cumulative duration of pioglitazone use was treated as a continuous variable, the hazard ratio was significant, 0.987 (95% confidence interval: 0.976–0.998) for every 1 month of use.

Table 3 shows the results of the subgroup analyses conducted in ever users and never users of metformin, separately. Significant *p*-values were only obtained among patients who had never used metformin. Among metformin never users, the overall hazard ratio suggested a 50% risk reduction and a dose-response relationship could be observed in both the tertile analysis and in the analysis that treated cumulative duration as a continuous variable. The tertile analysis in never users of metformin showed a significantly and remarkably lower risk in patients who had used pioglitazone for a cumulative duration of more than approximately 20 months in the third tertile.

Table 4 shows the joint effects of pioglitazone and the major risk factors of dementia. All models showed that the hazard ratios were lowest in patients who used pioglitazone and without the major risk factors of dementia, when compared to patients with the risk factors but without pioglitazone use (though not significant for the model in the analysis of pioglitazone and head injury). While *p*-trends (<0.01) were significant in all models, the *p*-interaction (>0.05) did not favor any significant interaction between pioglitazone and the major risk factors.

## 4. Discussion

The findings suggested that pioglitazone use in patients with type 2 diabetes mellitus was associated with a significantly lower risk of dementia (Table 2), especially when the patients had not been treated with metformin and had used pioglitazone for more than 20 months (Table 3). No interaction was observed between pioglitazone and major risk factors of dementia including stroke, hypoglycemia, head injury and Parkinson’s disease (Table 4).

Although the mechanisms of a reduced risk of dementia associated with pioglitazone use require more investigation, some biological actions of pioglitazone on the brain could explain its beneficial effect. PPARγ is expressed in brain tissue [18] and pioglitazone (a PPARγ agonist) may cross the blood-brain-barrier [19]. Knockdown of the PPARγ gene affects the expression of several genes associated with Alzheimer’s disease, suggesting that pioglitazone may regulate the transcription of genes related to Alzheimer’s disease and may potentially affect the risk of dementia [20]. Pioglitazone may alleviate insulin resistance, reduce Aβ synthesis, inhibit neuroinflammation and improve energy utilization and lipid metabolism in the brain [1]. However, because brain concentration of pioglitazone is limited by P-glycoprotein, a drug efflux transporter, and (+)-pioglitazone is more resistant to this efflux transporter and accumulates in higher concentrations in the brain tissue than (−)-pioglitazone does [8], this stereoselectivity on brain penetration of pioglitazone may help develop more efficient compound of pioglitazone to be used as a preventive or therapeutic agent for dementia.

Metformin also crosses the blood-brain-barrier [21] and has been shown to reduce the risk of dementia, likely through different mechanisms [11]. The findings support a beneficial effect of pioglitazone independent of metformin (Table 2) because ever users and never users of pioglitazone were well matched in metformin use (Table 1). However, the subgroup analyses also suggested that the beneficial effect of pioglitazone was greater in patients who had never been treated with metformin (Table 3), even though no interaction was found in secondary analysis.

Stroke is a major risk factor of dementia [22] and pioglitazone may reduce the risk of stroke in either diabetes patients [23] or in non-diabetes patients with ischemic stroke or transient ischemic attack and insulin resistance [24]. To further examine whether the effect of pioglitazone might be related to a stroke event, secondary analyses were conducted to estimate the overall hazard ratios in patients with a history of stroke and in those without a stroke diagnosis. The respective hazard ratios for ever versus never users of pioglitazone were 0.608 (0.418–0.883) and 0.870 (0.581–1.304), suggesting that the protective effect of pioglitazone on dementia might be greater in patients with a history of stroke. Therefore, pioglitazone may be a good choice for the management of hyperglycemia in diabetes patients with stroke. Similarly, the overall hazard ratios for patients with and without any of the other three major risk factors of dementia (i.e., hypoglycemia, head injury and Parkinson’s disease) in secondary analyses were 0.684 (0.379–1.234) and 0.725 (0.532–0.988), respectively. However, the analyses consistently suggested a lack of significant interaction between pioglitazone and the major risk factors of dementia (Table 4).

It is important to point out that the design of the present study was aimed at mimicking a clinical trial that evaluated the effect of pioglitazone in comparison to a placebo (i.e., ever versus never users of pioglitazone). Therefore, the findings could be interpreted as a potentially protective effect of pioglitazone on the risk of dementia. On the other hand, the findings of the study by Lu et al. should not be concluded as a protective effect of pioglitazone on dementia [10]. At most, it could be interpreted as a better effect of pioglitazone in patients who failed to be treated with metformin in comparison to patients who were given sulfonylureas on top of metformin [10].

The study has merits of using a nationwide database that covers >99% of the Taiwan’s population, so that the findings can be readily generalized to the whole population. The use of medical records significantly reduced biases related to self-reporting. Detection bias due to different socioeconomic status was less likely because of the low drug cost-sharing in the NHI which can also be waived in patients with low-income household, in veterans and when receiving prescription refills for chronic disease.

The study limitations may include a lack of measurement data of some confounders like anthropometric factors, dietary pattern, nutritional status, lifestyle, smoking, alcohol drinking, family history and genetic parameters (e.g., Apo E4 genotype). Furthermore, we did not have biochemical data of blood levels of glucose and insulin and indicators of insulin resistance or β-cell function, such as Homeostatic Model Assessment for Insulin Resistance (HOMA-IR) and HOMA-β, for analyses.

In summary, the present study supports a beneficial effect of pioglitazone on the prevention of dementia in patients with type 2 diabetes mellitus. The beneficial effect is greater in patients who have not been treated with metformin and have been treated with pioglitazone for more than 20 months. There are no significant interactions between pioglitazone and major risk factors of dementia including stroke, hypoglycemia, head injury and Parkinson’s disease. The findings give rationale for conducting clinical trials to prove such an effect. Given that both metformin and pioglitazone do not cause hypoglycemia and both may potentially reduce the risk of dementia, pioglitazone could be considered as a second-line therapy for patients with type 2 diabetes mellitus after metformin, in patients who do not tolerate the side effects of metformin or in those who are not indicated for metformin use, especially in those who are at a high risk of developing dementia.

## Figures and Tables

**Table 1:** Characteristics in never and ever users of pioglitazone.

Variables	Never Users		Ever Users		Standardized Difference
	(*n* = 11,011)		(*n* = 11,011)		
	*n*	%	*n*	%	
Demographic data					
Age (years)	58.75	10.10	58.71	9.71	−0.38
Sex (men)	6218	56.47	6294	57.16	1.37
Diabetes duration (years)	6.46	2.78	6.41	2.58	−1.94
Occupation					
I	4334	39.36	4403	39.99	
II	2607	23.68	2593	23.55	−0.21
III	2074	18.84	2043	18.55	−0.78
IV	1996	18.13	1972	17.91	−0.60
Living region					
Taipei	4324	39.27	4341	39.42	
Northern	1134	10.30	1115	10.13	−0.62
Central	1790	16.26	1759	15.97	−0.79
Southern	1390	12.62	1344	12.21	−1.24
Kao-Ping and Eastern	2373	21.55	2452	22.27	1.66
Major comorbidities associated with diabetes mellitus					
Hypertension	8927	81.07	8810	80.01	−2.64
Dyslipidemia	9507	86.34	9477	86.07	−0.72
Obesity	716	6.50	725	6.58	0.31
Diabetes-related complications					
Nephropathy	2704	24.56	2600	23.61	−2.22
Eye disease	3766	34.20	3749	34.05	−0.33
Stroke	2321	21.08	2306	20.94	−0.37
Ischemic heart disease	4437	40.30	4383	39.81	−0.91
Peripheral arterial disease	2427	22.04	2492	22.63	1.43
Other major risk factors of dementia					
Head injury	366	3.32	337	3.06	−1.59
Parkinson’s disease	131	1.19	130	1.18	−0.10
Hypoglycemia	301	2.73	328	2.98	1.38
Potential risk factors of cancer					
Chronic obstructive pulmonary disease	4644	42.18	4690	42.59	0.77
Tobacco abuse	464	4.21	458	4.16	−0.25
Alcohol-related diagnoses	615	5.59	628	5.70	0.47
Antidiabetic drugs					
Insulin	360	3.27	343	3.12	−0.92
Sulfonylureas	7892	71.67	7855	71.34	−0.65
Metformin	8359	75.91	8338	75.72	−0.27
Meglitinide	728	6.61	739	6.71	0.50
Acarbose	1432	13.01	1500	13.62	1.81
Medications commonly used in diabetes patients					
Angiotensin converting enzyme inhibitors/angiotensin receptor blockers	8028	72.91	8013	72.77	−0.24
Calcium channel blockers	6043	54.88	6011	54.59	−0.54
Statins	8226	74.71	8274	75.14	1.03
Fibrates	5054	45.90	5042	45.79	−0.18
Aspirin	6223	56.52	6266	56.91	0.86

Age and diabetes duration are shown in mean and standard deviation.

**Table 2:** Incidence rates and hazard ratios of dementia by pioglitazone exposure.

Pioglitazone Use	*n*	*N*	Person-Years	Incidence Rate (Per 100,000 Person-Years)	HR	95% CI	*p*-Value
Never users	123	11,011	28,378.04	433.43	1.000		
Ever users	91	11,011	29,612.81	307.30	0.716	(0.545–0.940)	0.0163
Tertiles of cumulative duration of pioglitazone therapy (months)							
Never users	123	11,011	28,378.04	433.43	1.000		
<11.0	32	3636	9537.25	335.53	0.806	(0.544–1.193)	0.2809
11.0–19.6	27	3613	9746.22	277.03	0.654	(0.430–0.994)	0.0467
>19.6	32	3762	10,329.34	309.80	0.694	(0.469–1.026)	0.0670
Cumulative duration of pioglitazone therapy treated as a continuous variable							
For every 1-month increment of pioglitazone use					0.987	(0.976–0.998)	0.0246

*n*: incident cases of dementia, *N*: cases followed, HR: hazard ratio, CI: confidence interval.

**Table 3:** Subgroup analyses with regards to metformin use for incidence rates and hazard ratios of dementia by pioglitazone exposure.

Metformin Use/Pioglitazone Use	*n*	*N*	Person-Years	Incidence Rate (Per 100,000 Person-Years)	HR	95% CI	*p*-Value
Metformin ever users							
Pioglitazone never users	85	8359	21,706.67	391.58	1.000		
Pioglitazone ever users	66	8338	22,445.80	294.04	0.802	(0.580–1.109)	0.1822
Tertiles of cumulative duration of pioglitazone therapy (months)							
Never users	85	8359	21,706.67	391.58	1.000		
<11.0	23	2781	7319.59	314.23	0.874	(0.549–1.392)	0.5719
11.0–19.6	6	2732	7379.70	81.30	0.603	(0.352–1.032)	0.0649
>19.6	27	2825	7746.51	348.54	0.915	(0.591–1.417)	0.6920
Cumulative duration of pioglitazone therapy treated as a continuous variable							
For every 1-month increment of pioglitazone use					0.991	(0.978–1.004)	0.1772
Metformin never users							
Pioglitazone never users	38	2652	6671.38	569.60	1.000		
Pioglitazone ever users	25	2673	7167.01	348.82	0.494	(0.284–0.857)	0.0121
Tertiles of cumulative duration of pioglitazone therapy (months)							
Never users	38	2652	6671.38	569.60	1.000		
<11.0	9	855	2217.66	405.83	0.588	(0.272–1.273)	0.1778
11.0–19.6	11	881	2366.52	464.82	0.690	(0.338–1.409)	0.3084
>19.6	5	937	2582.84	193.59	0.265	(0.102–0.688)	0.0064
Cumulative duration of pioglitazone therapy treated as a continuous variable							
For every 1-month increment of pioglitazone use					0.974	(0.952–0.998)	0.0306

*n*: incident cases of dementia, *N*: cases followed, HR: hazard ratio, CI: confidence interval.

**Table 4:** Joint effects between pioglitazone and major risk factors of dementia.

Major Risk Factor/Pioglitazone Use	*n*	*N*	Person-Years	HR	95% CI	*p* Value
Stroke (+)/Pioglitazone (−)	72	2321	5986.79	1.000		
Stroke (+)/Pioglitazone (+)	46	2306	6153.77	0.617	(0.425–0.895)	0.0110
Stroke (−)/Pioglitazone (−)	51	8690	22,391.25	0.317	(0.217–0.463)	<0.0001
Stroke (−)/Pioglitazone (+)	45	8705	23,459.04	0.271	(0.183–0.402)	<0.0001
Hypoglycemia (+)/Pioglitazone (−)	13	301	789.68	1.000		
Hypoglycemia (+)/Pioglitazone (+)	11	328	869.14	0.775	(0.346–1.737)	0.5356
Hypoglycemia (−)/Pioglitazone (−)	110	10,710	27,588.36	0.430	(0.239–0.773)	0.0048
Hypoglycemia (−)/Pioglitazone (+)	80	10,683	28,743.67	0.304	(0.167–0.554)	0.0001
Head injury (+)/Pioglitazone (−)	6	366	926.96	1.000		
Head injury (+)/Pioglitazone (+)	8	337	891.60	1.365	(0.472–3.951)	0.5655
Head injury (−)/Pioglitazone (−)	117	10,645	27,451.08	0.954	(0.417–2.180)	0.9102
Head injury (−)/Pioglitazone (+)	83	10,674	28,721.21	0.652	(0.283–1.502)	0.3149
Parkinson’s disease (+)/Pioglitazone (−)	8	131	330.96	1.000		
Parkinson’s disease (+)/Pioglitazone (+)	7	130	351.72	0.768	(0.276–2.132)	0.6119
Parkinson’s disease (−)/Pioglitazone (−)	115	10,880	28,047.08	0.396	(0.191–0.822)	0.0129
Parkinson’s disease (−)/Pioglitazone (+)	84	10,881	29,261.09	0.282	(0.135–0.591)	0.0008
Any of the four (+)/Pioglitazone (−)	80	2779	7168.34	1.000		
Any of the four (+)/Pioglitazone (+)	52	2784	7430.66	0.635	(0.447–0.903)	0.0113
Any of the four (−)/Pioglitazone (−)	43	8232	21,209.70	0.282	(0.192–0.415)	<0.0001
Any of the four (−)/Pioglitazone (+)	39	8227	22,182.15	0.249	(0.167–0.372)	<0.0001

*n*: incident cases of dementia, *N*: cases followed, HR: hazard ratio, CI: confidence interval; For all models: *p*-trend < 0.01 and *p*-interaction > 0.05.
